# Attitudes Regarding Participation in a Diabetes Screening Test among an Assyrian Immigrant Population in Sweden

**DOI:** 10.1155/2016/1504530

**Published:** 2016-12-19

**Authors:** Susanne Andersson, Veronika Karlsson, Louise Bennet, Klas Fellbrant, Margareta Hellgren

**Affiliations:** ^1^School of Health and Education, University of Skövde, Building G, Floor 3, Högskolevägen 1, 541 28 Skövde, Sweden; ^2^Department of Health Sciences, University West, Trollhättan, Sweden; ^3^Center for Primary Health Care Research, Family Medicine, Department of Clinical Sciences, Lund University, Malmö, Sweden; ^4^Family Medicine, Department of Primary Health Care, Skövde, Sweden; ^5^Institute of Medicine, Department of Primary Health Care, Sahlgrenska Academy, University of Gothenburg, Gothenburg, Sweden

## Abstract

Immigrants from the Middle East have higher prevalence and incidence of type 2 diabetes (T2D) compared with native Swedes. The aim of the study was to describe and understand health beliefs in relation to T2D as well as attitudes regarding participation in a screening process in a local group of Assyrian immigrants living in Sweden. A qualitative and quantitative method was chosen in which 43 individuals participated in a health check-up and 13 agreed to be interviewed. Interviews were conducted, anthropometric measurements and blood tests were collected, and an oral glucose tolerance test was performed. In total, 13 of the 43 participants were diagnosed with impaired glucose metabolism, 4 of these 13 had TD2. The interviewed participants perceived that screening was an opportunity to discover more about their health and to care for themselves and their families. Nevertheless, they were not necessarily committed to taking action as a consequence of the screening. Instead, they professed that their health was not solely in their own hands and that they felt safe that God would provide for them. Assyrians' background and religion affect their health beliefs and willingness to participate in screening for TD2.

## 1. Introduction

The rationale behind this study is that screening in high-risk populations is considered to be both cost-saving for society [1] and of great importance for the individuals concerned. Such a population is, for instance, immigrants from the Middle East who have higher prevalence of type 2 diabetes (T2D) compared with native Swedes [2]; in addition, they have a higher risk for cardiovascular disease [3]. The prevalence of T2D is estimated to increase not only in Sweden but also worldwide as a result of the adoption of a more sedentary lifestyle and an increased intake of energy-dense food [4]. Further, individuals from the Middle East seem to be genetically highly susceptible to T2D [5], presenting a serious problem both at an individual level and at a societal level. It is important to diagnose T2D because of the expected large number of unreported cases [6] and its insidious course [7]. Moreover, it is preceded by impaired glucose tolerance (IGT) or impaired fasting glucose (IFG) with vague symptoms [8, 9] but still with an increased risk of cardiovascular disease. Even if individuals with IGT have a sixfold higher risk of progression to T2D compared to individuals with normal glucose tolerance [10], landmark studies have shown that lifestyle interventions can efficiently prevent progression to T2D in these cases [11]. In addition, screening leads to early identification and treatment of individuals with T2D and it may, therefore, prevent complications such as cardiovascular disease and microvascular complications, nephropathy, retinopathy, and neuropathy [12]. Accordingly, screening for T2D is important, particularly in populations at high risk. To create adequate screening programs, we need information on health beliefs and screening in relation to T2D in this particular group.

### 1.1. Background

Assyrians are Christian Semitic people who generally define themselves through their common culture, religion, and origin. They originally come from Iraq, Syria, Turkey, Lebanon, and Jordan. Assyrians have immigrated to Sweden over the course of the last forty years. Today, there are an estimated 120,000 Assyrians in Sweden, yet the current mass migration of Assyrians to Sweden means that Assyrians will constitute an increasing proportion of the Swedish population. As they represent a high-risk group for diabetes, it is important that the Swedish health care system and the community gain a better understanding of the cultural and social aspects of this group. This knowledge will have a significant impact on their future ability to maintain good health.

Previous studies have determined that areas of socioeconomic deprivation exhibit a correlation with the incidence of T2D and that the prevalence of T2D, IGT, and IFG and risk factors for T2D are likely to be higher in immigrant-dense neighborhoods [4]. Thus, the increased risk of T2D cannot be explained only by changes in lifestyle or other well-known risk factors [2]. A report from the International Diabetes Federation [13] showed that countries located in the Middle East have high prevalence of T2D and when people from these countries immigrate to Sweden, the estimated risk of developing T2D is two to three times higher compared to the native population in Sweden [14, 15]. Nonwestern immigrants show poorer glycemic control than native Swedes in spite of earlier pharmacological treatment and frequent appointments [16]. For instance, immigrants from Iraq and Turkey living in Sweden are shown to have a higher risk of insulin resistance compared to native Swedes [14, 17]. In addition, a study by Hjörleifsdottir-Steiner et al. concluded that immigration, per se, is associated with an increased risk of T2D [5].

### 1.2. Aim

The aim of the study was to describe and understand health beliefs in relation to T2D as well as attitudes regarding participation in a screening process in a local group of Assyrian immigrants living in Sweden.

## 2. Methods

### 2.1. Design

This study was performed using qualitative and quantitative methodology in a primary health care setting [18]. The data collection started with an invitation to all members of a congregation aged between 30 and 75 years who had not previously been diagnosed with diabetes mellitus (*n* = 100) in the Assyrian congregation in Skövde. We offered them a health examination with a focus on glucose metabolism. A total of 43 individuals agreed to participate in the health examination, 13 of which (eight women and five men) were interviewed concerning the process of screening and their health beliefs. Both men (*n* = 5) and women (*n* = 8), as well as individuals of different ages, participated in the interviews; we, therefore, consider them to be representative of the population of an Assyrian congregation.

### 2.2. Subjects and Context

The target group of this cross-sectional study was Assyrians aged between 30 and 75 years, without prior diagnosis of diabetes. In a medium-sized town in the southwest of Sweden (50,000 inhabitants), there is a well-assimilated Assyrian congregation willing to participate in a study concerning diabetes and diabetes prevention. To get in touch with the Assyrian community, two local churches were contacted. After a church service in each congregation, the aim and design of the study were presented to the congregation and written information was distributed in both Swedish and Arabic. In a subsequent church visit, those who wished to participate were given a health check-up appointment at the primary health care center and also invited to participate in an interview. In total, 43 participants agreed to a health check-up and 13 of these agreed also to be interviewed.

### 2.3. Interviews

In order to understand the participants' beliefs about their health in relation to their increased risk of developing T2D, they were asked to take part in an interview. The interviews took place prior to the health examination, which meant that, at the time of the interview, the participants had no knowledge of the results of the assessments or the glucose tolerance test. A total of eight women and five men agreed to be interviewed. The semistructured interviews were recorded on digital tape and transcribed verbatim. The following are the interview questions in a typical interview that lasted approximately 15 min. 

*Interview Questions*

*Initial Question*

- You have chosen to participate in this study aimed to investigate if you have any health risks such as high blood sugar. What is it that has made you agree to participate in this survey?

*Follow Up Questions*

- What do you think of the prospect that you may have high blood sugar?
- Who and/or what prompted you to come here today?
- How do you feel about participating in the study?
- Have you previously considered the possibility that you might have high blood sugar levels?
- Do you know anyone else who has diabetes or elevated blood sugar levels?
- What would it mean for you if it turns out that you have high blood sugar?

The interviews were conducted by two of the authors (SA and VK) and carried out in the autumn of 2013, with the help of a volunteer interpreter in required cases (8 out of 13 participants). The interpreter was not previously known to the participants or to the researchers.

### 2.4. Interpretation and Analysis

The analysis of the interviews was conducted by means of qualitative content analysis [19]. According to Graneheim and Lundman, it is appropriate to use qualitative content analysis when one receives a smaller amount of data (text) in the form of interviews. Once all the interviews were transcribed, they were read through in their entirety several times and the research questions were directed to the text. Meaning units (units of text) corresponding to the aim were then selected and divided into condensed meaning units, subthemes, and themes (Table 1). The authors maintained an ongoing dialogue during the analysis to ensure consistency in interpretation and descriptions [19].

### 2.5. Physical Examination and Blood Samples

The quantitative part of the study was performed by collecting data where all participants were examined in the morning with an oral glucose tolerance test (OGTT) after an overnight fast. Blood tests were drawn for HbA1c, total cholesterol, LDL, HDL, and triglycerides. Type 2 diabetes was diagnosed according to World Health Organization [20]. Questionnaires concerning lifestyle factors were completed. Approximately one week later, a physical examination was performed by a physician who also collected anthropometric data: waist circumference (WC) measured to the nearest cm at the widest part between the lower chest and spina iliaca anterior superior, body weight measured to the nearest 0.1 kg on a calibrated scale, body height measured to the nearest cm (light indoor clothes, no shoes), and sagittal diameter measured in a supine position as the distance between the back and the highest point of the abdomen. Blood pressure (mmHg) was measured in a sitting position after five-minute rest and a nonfasting plasma glucose was examined. The test results were discussed with the participants and, in cases of pathologies such as diabetes (Table 2) or a blood pressure ≥140/90, the participants were referred to their local health care center for further examination. Epidemiological data were analyzed with SPSS18.0 and reported as means with standard deviation.

### 2.6. Basic Characteristics of the Background Population

A total of 43 members of the Assyrian Orthodox Church participated in the study and 13 of these individuals accepted the interview request. Of the participants, 14 were born in Iraq, 12 in Turkey, 8 in Syria and Lebanon, respectively, and one in Jordan. The participants had been in Sweden for varying lengths of time; the earliest immigrant came in 1975 and the last one in 2013.

In total, 13 (30.2%) of the 43 participants had impaired glucose metabolism, whereof 4 (9%) were diagnosed with type 2 diabetes. Mean waist in men was 100 cm (SD 15.0) and in women 91 cm (SD 11.4) and mean HDL in men was 1.2 mmol/L (SD 0.3) and in women 1.7 mmol/L (0.3). Smoking was more common in men (23%) than in women (12%), yet there was no significant difference (*p* = 0,293). Among those with normal glucose tolerance, 19% were smokers and among those with impaired glucose tolerance, 15% were smokers.

### 2.7. Ethical Consideration

Approval for this study was granted by the Regional Ethical Review Board of the University of Gothenburg, Sweden (Diary number 426-13); furthermore, the study conforms to the principles outlined in the Declaration of Helsinki [21]. The participants received written and oral information in both Swedish and Arabic and informed consent was obtained. The participants who agreed to participate in the health survey were also asked to participate in an interview. It was stressed that participation in the interview was completely voluntary and that it was possible to only participate in the health examination. The collected data were classified to prevent participants from being identified. In addition, the collected material was used only for the purpose of this study.

## 3. Results

The results are structured in correlation to the research questions.

### 3.1. Results from Interviews

In the following text, the text in italics refers to the themes that emerged during the analysis.

#### 3.1.1. Willingness to Know

When asked after the church service to participate in the screening, there were 43 participants who expressed an interest and 13 agreed to participate in an interview. One reason mentioned was a desire to know whether they were in good health, particularly in relation to possible diabetes. Diabetes was often discussed in connection with the meetings of the church as many had a relative or a close acquaintance with the condition and there was awareness that their Assyrian migrant background posed an increased risk of developing T2D.

*“When one gets it, then it's good to know, it's worse not knowing it.” (woman, interview 12)*

Another aspect was the desire to confirm that they were in good health. They believe that health is important, both for your own sake and for the sake of the family. Health was described as a major and important part of life, a truly fundamental condition. There was a desire to know more and to be able to do something to achieve better health.

#### 3.1.2. The Decision Is Not Only Their Own

The participants' strong faith in God and their religion (Christianity) influence their perception of illness and in what way they are affected. Whether they will suffer from a disease like T2D or not is beyond their control; they may not always be able to influence their health through their own actions, such as diet and exercise; they believe the responsibility lies with God, in accordance with their religious faith. They leave their lives in God's hands without worrying too much about their health, unless they receive other symptoms. When they are affected by a disease, it is deemed natural and something they just have to accept.

*“It is from God. I can do nothing” (woman, interview 2)*

Presence of illness or health is not something determined by the individuals. The participants argue that it is not possible to prevent or affect the onset of T2D by eating in a healthy manner or by being physically active. Habits are hard to break, but if the disease does strike them, they seem to be willing to make changes.

*“We take the diet and then we take care of ourselves and take care of our life” (man, interview 6)*

There was a desire among participants to adjust their lifestyles through dietary changes and medication and in case of a manifest diagnosis of T2D it was emphasized in order to achieve good health.

#### 3.1.3. Extended Responsibility for Other People

Reasons given for a positive attitude to screening were concerns not only about their own health but also for the health of their children. The extended responsibility for other people's health was important, but most important was to take responsibility for their own family and their health.

*“Of course, of course you think about it (health) because when one has four kids then you have obligations towards them” (woman, interview 9)*

The health of their children had priority over the participants' own well-being. Participants described an obligation towards their children because of concerns about the high heritability of T2D among their ethnic group. A willingness to understand and the need for explanation prompted a reflection as to why they, as a group, are affected. Issues such as the intake of sugar, unhealthy habits, less physical activity, and how these interact were also considered.

*“Because it is hereditary you are concerned that your children will be affected, for your wife, family, I am afraid of this” (man, interview 8)*

The attitudes of relatives, friends, and others in their neighborhood who have T2D and how this has affected their health and well-being were major contributing factors for participation in screening. Other important arguments to participate were aiding research and helping their community. They also expressed a responsibility towards Sweden and its citizens.

*“And I want that (participation in the screening) for my people” (woman, interview 1)*

The participants expressed a positive attitude regarding participation in the screening process. Screening was seen as an opportunity to find out about their own health status and as an extended responsibility to care for their families, friends, and even the nation. Cultural and religious beliefs were referred to as a motive for participating in screening for type 2 diabetes and to their attitude towards changing their lifestyle as a result of the test. In addition, there is an awareness of the fact that, as nonwestern immigrants, they constitute a risk group; this added to their willingness to partake in the screening.

## 4. Discussion

This study aimed to describe health beliefs in relation to T2D as well as attitudes regarding participation in a screening process in a local group of Assyrian immigrants living in Sweden. Interviews can often lead to an increased level of knowledge of a subject [19] and have the potential to describe health beliefs. The study confirmed the high prevalence of abnormal glucose tolerance in immigrants from the Middle East [14] with many previously undiagnosed individuals, 9% of which with T2D. The quantitative part of the study made an interesting background to the qualitative part that illuminated the health beliefs of the population studied and their reason to participate in screening.

The participation rate in screening procedures among Swedes varies considerably. While a health examination for blood pressure and diabetes engaged 66% of the target population in a major city, 81% in a small city in the countryside were willing to participate [22]. In a screening program for type 2 diabetes targeting 10.000 individuals performed in the same city as the present study, the response rate was 66% [23]. There are no data, as far as we know, concerning participation rates in screening programs for diabetes addressing immigrants. However, there are data concerning breast cancer, where regular screening has been used for many years, and while the screening engages 73% of the target population, female immigrants had 1.7 times as high risk to refrain from screening compared to native Swede women [24].

In this qualitative study, we found that the participants' decision to take part in the diabetes screening process was based on their health beliefs and the desire to know more about their personal health. Knowledge about the increased risk for onset of T2D [14] for Assyrian immigrants, as well as for their own health, was high and the participants wanted to find out about metabolic risks in order to prevent the disease. These results are congruent with earlier research [25] showing that it can be distressing to have an increased risk of developing T2D. This feeling may naturally change over time.

The Assyrian population living in Sweden has the tendency to develop T2D earlier compared to native Swedes [13, 16, 26]. The participants in this study had an awareness regarding the increased risk of T2D among immigrants. For example, they often discussed the subject in meetings in church. These results are in line with research by Bennet et al. [2], where immigrants from Iraq have participated in screening to a greater extent compared to native Swedes. Reasons for this may be the fact that they have more time to participate due to not only higher rates of unemployment but also the knowledge of the increased risk of T2D. The present study shows that the participants' religion and their faith in God affected the way they experienced living with the increased risk for T2D. The results are in line with earlier research describing God's omnipresence and importance in the lives of religious people, irrespective of whether they are Syrian Orthodox Christian or Muslim [27]. The participants in the study claim that God determines who will suffer from illness. This view is not considered to be in conflict with a personal responsibility to avoid the risk of developing T2D and as a consequence adopt a healthier lifestyle. The results of this study also imply that the participants are not necessarily motivated to change their lifestyle to counter the risk of T2D. Thus, religion and its relation to health and health beliefs are considered important determining factors among the Middle East women [28]. The participants in this study ultimately leave their lives in the hands of God and suggest there may be a meaning associated with disease. Being in God's hands means to be consoled and living in a relationship of trust in God for oneself and others [29]. This belief may influence the participation in a screening process. The participants that were interviewed in this study had all agreed to participate in the screening; however, the results of the interviews may partly explain why many at risk of developing T2D declined the opportunity to have a health examination.

These findings are also supported by earlier research [28, 30, 31] showing that religion plays a prominent role in family relationships and that faith contributes to a strong cohesion within the family. The high prevalence of smoking reported in the questionnaires is also in line with traditions in the Middle East and has not been influenced by the knowledge of the deleterious effects of smoking. This may be due to the perspective that everything is in the hands of God or possibly due to a lack of knowledge of the consequences of smoking.

According to Käppeli [32], religiousness has great potential as a resource and caregivers should be aware of this and give proper support, which this study also emphasizes.

### 4.1. Limitations and Strengths

A limitation is the small study population with only 43 participants and also the number of interviews. About 50% of the possible participants agreed to take part of the study. Our focus was the reasons for participating in a screening program and we can only speculate about the reasons for not participating. Many of the participants mentioned that their fate is dependent on God's will, a fact that is likely to influence their willingness to participate in a screening program, and there may also be a denial of the individual risk for type 2 diabetes. Also, only thirteen of the participants agreed to be interviewed, most likely because an interview is time-consuming; the reasons why people do not participate need to be further explored.

We chose to recruit participants for the study through the church; this was based on the fact that church is a common place for all Assyrians to meet. As a consequence of this method, we cannot express any conclusions about other ethnic groups from the Middle East and consequently our results are only valid for this particular group. However, the results are likely to represent about 120.000 individuals in Sweden. Some of the participants who intended to take part declined the researchers' request to participate in the study, which undeniably limits the validity of the study. This study consisted of 13 interviews that lasted between 10 and 18 minutes. Although the interviews were limited in length and not always in-depth, the data collected were deemed sufficiently rich for an analysis on a descriptive level. Another limitation was the fact that the invited participants were well-assimilated immigrants, which limits the generalizability of the findings to the Assyrian population in Sweden in general.

The interviews were read several times by two of the authors to ensure immersion in the data, which resulted in a manifest analysis. The researcher in an interview is regarded as a cocreator of the text and thus cannot be independent of the outcome [19]. The interviewers have extensive experience as nurses and are accustomed to communicating with patients, which may have been an advantage. As a result, the interviews are more in-depth and the participants have had the opportunity to reflect on their answers. The interpreter was unknown to both the participants and the interviewers and was a person who knew the language and culture well, which should be considered as a strength.

To increase the participation rate in the study and to be able to get as valid answers as possible, the researchers made contact with the congregation on several occasions before the interviews took place. While this may be considered as a strength, it may have influenced the attitude towards screening.

## 5. Conclusion

The results showed that the health beliefs of the Assyrian population studied here affected the willingness to participate in a health check-up. The participants expressed many reasons for taking part in the health check-up; they wanted to participate for both their own sake and for the sake of their family as well as that of society. Participants showed some ambiguity in their attitude in relation to their faith in God and destiny and the ability to influence the life habits. Concerns about heredity and an obligation towards children and family turned out to be major contributing factors for participating in the screening.

## 6. Implications

Background and religion affect the health beliefs and willingness to participate in screening for T2D in Assyrian immigrants. Their health perceptions are based on their religious background and their belief in an inevitable fate. This is something the caregiver needs to be aware of when such examinations are offered. This belief also affects their willingness to implement lifestyle interventions, since eventual diseases are deemed to be God's will. Culturally appropriate programs are required considering screening suitable for this population [33]. These results can be viewed as new knowledge regarding persons with Assyrian background, both the high prevalence of abnormal glucose metabolism and the reasons for participating in screening for T2D. It is particularly important to capture the motive for accepting to participate in a health check-up process and to consider the different factors emphasized in this study.

## Figures and Tables

**Table 1:** Examples of meaning units, condensed meaning units, subtheme, and theme in the analysis.

Meaning units	Condensed meaning units	Subtheme	Theme
“It is from God. I cannot do anything about it”	My health is in the hands of God	In the hands of God	The decision is not only their own
“God determines who suffers from illness, not humans”	The responsibility lies with God	God decides	

**Table 2:** Anthropometric characteristics of the surveyed population and outcome regarding glucose metabolism.

Variable	Data (*n* = 43)
Age (years)	51 ± 10
Men (*n*)	18
Women (*n*)	25
Type 2 diabetes (*n*)	4
Impaired glucose tolerance (*n*)	3
Impaired fasting glucose (*n*)	6
Smoking (%)	19
Blood pressure syst (mmHg)	122 ± 17
Blood pressure dia (mmHg)	72 ± 10
Cholesterol (mmol/L)	5,1 ± 1,1
Body mass index (kg/m^2^)	29 ± 5

## References

[1] Tamayo T., Rosenbauer J., Wild S. H. (2014). Diabetes in Europe: an update. *Diabetes Research and Clinical Practice*.

[2] Bennet L., Groop L., Franks P. W. (2014). Ethnic differences in the contribution of insulin action and secretion to type 2 diabetes in immigrants from the Middle East compared to native Swedes. *Diabetes Research and Clinical Practice*.

[3] Bennet L., Agardh C.-D., Lindblad U. (2013). Cardiovascular disease in relation to diabetes status in immigrants from the Middle East compared to native Swedes: a cross-sectional study. *BMC Public Health*.

[4] Bennet L., Johansson S.-E., Agardh C.-D. (2011). High prevalence of type 2 diabetes in Iraqi and Swedish residents in a deprived Swedish neighbourhood—a population based study. *BMC Public Health*.

[5] Hjörleifsdottir-Steiner K., Satman I., Sundquist J., Kaya A., Wändell P. (2011). Diabetes and impaired glucose tolerance among Turkish immigrants in Sweden. *Diabetes Research and Clinical Practice*.

[6] Bennet L., Johansson S.-E., Agardh C.-D. (2011). High prevalence of type 2 diabetes in Iraqi and Swedish residents in a deprived Swedish neighbourhood—A Population Based Study. *BMC Public Health*.

[7] World Health Organization (2010). *Chapter 1-Burden: Mortality, Morbidity and Risk Factors*.

[8] Andersson S., Ekman I., Lindblad U., Friberg F. (2011). Perceived symptoms in people living with impaired glucose tolerance. *Nursing Research and Practice*.

[9] Leahy J. L. (2005). Pathogenesis of type 2 diabetes mellitus. *Archives of Medical Research*.

[10] Gerstein H. C., Santaguida P., Raina P. (2007). Annual incidence and relative risk of diabetes in people with various categories of dysglycemia: a systematic overview and meta-analysis of prospective studies. *Diabetes Research and Clinical Practice*.

[11] Tuomilehto J., Lindström J., Eriksson J. G. (2001). Prevention of type 2 diabetes mellitus by changes in lifestyle among subjects with impaired glucose tolerance. *New England Journal of Medicine*.

[12] Waugh N., Scotland G., McNamee P. (2007). Screening for type 2 diabetes: literature review and economic modelling. *Health Technology Assessment*.

[13] IDF. Diabetes Atlas, International Diabetes Federation (Ed), 2013, http://www.idf.org/diabetesatlas

[14] Bennet L., Groop L., Lindblad U., Agardh C.-D., Franks P. W. (2014). Ethnicity is an independent risk indicator when estimating diabetes risk with FINDRISC scores: a cross sectional study comparing immigrants from the Middle East and native Swedes. *Primary Care Diabetes*.

[15] Wändell P. E., Johansson S. E., Gåfvels C., Hellénius M. L., de Faire U., Sundquist J. (2008). Estimation of diabetes prevalence among immigrants from the Middle East in Sweden by using three different data sources. *Diabetes & Metabolism*.

[16] Rawshani A., Svensson A.-M., Rosengren A., Zethelius B., Eliasson B., Gudbjörnsdottir S. (2015). Impact of ethnicity on progress of glycaemic control in 131 935 newly diagnosed patients with type 2 diabetes: a nationwide observational study from the Swedish national diabetes register. *BMJ Open*.

[17] Abdul-Ghani M. A., Jenkinson C. P., Richardson D. K., Tripathy D., DeFronzo R. A. (2006). Insulin secretion and action in subjects with impaired fasting glucose and impaired glucose tolerance: Results from the Veterans Administration Genetic Epidemiology Study. *Diabetes*.

[18] Polit F. D., Beck C. T. (2012). *Nursing Research: Generating and Assessing Evidence for Nursing Practice*.

[19] Graneheim U. H., Lundman B. (2004). Qualitative content analysis in nursing research: concepts, procedures and measures to achieve trustworthiness. *Nurse Education Today*.

[20] Alberti K. G. M. M., Zimmet P. Z. (1998). Definition, diagnosis and classification of diabetes mellitus and its complications. Part 1: diagnosis and classification of diabetes mellitus provisional report of a WHO consultation. *Diabetic Medicine*.

[21] Rickham P. (1964). Human experimentation. Code of ethics of the World Medical Association. Declaration of Helsinki. *British Medical Journal*.

[22] Andersson S., Ekman I., Friberg F., Daka B., Lindblad U., Larsson C. A. (2013). The association between self-rated health and impaired glucose tolerance in Swedish adults: a cross-sectional study. *Scandinavian Journal of Primary Health Care*.

[23] Hellgren M. I., Petzold M., Björkelund C., Wedel H., Jansson P.-A., Lindblad U. (2012). Feasibility of the FINDRISC questionnaire to identify individuals with impaired glucose tolerance in Swedish primary care. A Cross-sectional Population-based Study. *Diabetic Medicine*.

[24] Zackrisson S. http://malmo.se/download/18.4c0fed9713a59dfc9748000169/1383647140621/Mammografiscreening+i+Malm%C3%B6_Sophia+Zackrisson.pdf.

[25] Andersson S., Ekman I., Lindblad U., Friberg F. (2008). It's up to me! Experiences of living with pre-diabetes and the increased risk of developing type 2 diabetes mellitus. *Primary Care Diabetes*.

[26] Mansour A. A., Wanoose H. L., Hani I., Abed-Alzahrea A., Wanoose H. L. (2008). Diabetes screening in Basrah, Iraq: a population-based Cross-sectional Study. *Diabetes Research and Clinical Practice*.

[27] Hjelm K., Bard K., Nyberg P., Apelqvist J. (2003). Religious and cultural distance in beliefs about health and illness in women with diabetes mellitus of different origin living in Sweden. *International Journal of Nursing Studies*.

[28] Hjelm K. G., Bard K., Nyberg P., Apelqvist J. (2005). Beliefs about health and diabetes in men of different ethnic origin. *Journal of Advanced Nursing*.

[29] Fischer R. S., Nygren B., Lundman B., Norberg A. (2007). Living amidst consolation in the presence of God perceptions of consolation among the oldest old: the umeå 85+ study. *Journal of Religion, Spirituality & Aging*.

[30] Wallin A.-M., Ahlström G. (2010). From diagnosis to health: a cross-cultural interview study with immigrants from Somalia. *Scandinavian Journal of Caring Sciences*.

[31] Marks L. (2004). Sacred practices in highly religious families: Christian, Jewish, Mormon, and Muslim perspectives. *Family Process*.

[32] Käppeli S. (2000). Between suffering and redemption. *Scandinavian Journal of Caring Sciences*.

[33] Saha S., Leijon M., Gerdtham U. (2013). A culturally adapted lifestyle intervention addressing a Middle Eastern immigrant population at risk of diabetes, the MEDIM (impact of Migration and Ethnicity on Diabetes In Malmö): study protocol for a randomized controlled trial. *Trials*.

